# Cases Cured by Alexander Villers, M. D.

**Published:** 1895-04

**Authors:** 


### CASES CURED BY ALEXANDER VILLERS, M. D.

### From Archiv für Homöopathie.

[Translated by A. McNeil, M. D., San Francisco, Cal.]

### Case I.

A girl of sixteen has suffered since her fourth year from bleeding of the nose. Sometimes in her childhood this epistaxis would not occur for three days, and then it would be three or four times a day, and in the last two or three years no day passed without its recurrence, at least after her dinner. Formerly her face was red, then she would bleed for half an hour out of one nostril, and it would be followed by headache and thirst. She is always tired, of loose fibre, is troubled much from eructations, and has much palpitation of the heart. She rises in the morning unrefreshed, notwithstanding she has had a dreamless sleep, and always after her morning coffee there follows vomiting. Her menses are very profuse, always set in the fifteenth day, and continue eight, flowing night and day.

I prescribed Calcarea-carb.$^{cc}$, one drop to be given on the first, tenth, and twentieth day of her treatment. This was on the 19th of October.

When she again presented herself, on the 10th of November, she reported that she had only bled twice, and that on the two first days of her treatment. No nose-bleed occurred in all the rest of the time, except that after a blow on the nose a few drops fell, such as would happen to any healthy person. Her menses, which from her past experience would have set in on the fifteenth day of her treatment, did not appear till the typical twenty-eighth day, and only lasted four; but were of the same consistence and quantity as she has had since her twelfth year. From November 10th till the middle of February she took at intervals of fourteen days one drop Calcarea-carb.$^{cc}$, and during that time no hemorrhage occurred. Her periods were at the regular time, but always profuse. I had given to her mother, as a matter of precaution, China, with directions to give it to her daughter after every severe hemorrhage. But it had not been given, I am happy to say, as the clearness of the case would have been obscured thereby, and I could not have seen so accurately as I now do the effect of the high potency.

### Case II.

A full-formed woman, in the early forties, who was organically sound, had had, although her circumstances were brilliant, very much care and a great deal of hard work. She had frequent attacks of weakness, headache, changes in the amount of her menses, photophobia, palpitation of the heart; and mental depression in consequence has been the order of the day. All her physicians had diagnosed cerebral anæmia. I was not her attendant, but that of her husband. As I was frequently in the house she had fallen into the habit of more and more asking me for advice. I had heard much of these headaches, and had given her, at different times, Silicea, Ignatia, and Pulsatilla, according to the concomitants, and they had always done her good service, so that she had accumulated quite a stock of these remedies, and she always used them properly, according to her condition. But she now complains that none of them do her any good. This headache has continued fourteen days and has become intolerable. Although I questioned her several times, I could not learn anything characteristic, until I finally asked her whether or not she was better in the open air, when I received an astonishing reply. The headache is not so very violent, but its long continuance is unbearable. It is decidedly better, she says, in the open air; but she does not venture into the street because the sounds of the wagon-wheels are so horrible that she has the feeling as if every vehicle near her would run over her head, so loud did she hear the rolling of the wheels. And even in her house, which is on a tolerably quiet street, the passing vehicles affect her very unpleasantly through the double windows. Yet the noise of her children and of her household cause her very little annoyance. Calling to mind what I heard a colleague say at the World's Congress in Chicago, that a characteristic of Chinopodium was this peculiarity of hearing, oversensitiveness to the sound of carriage wheels, and although I knew nothing more of the drug, I gave her Chinopodium[6], which in three days removed the abnormal sensitiveness of hearing, and in the long interval that has elapsed she has not complained of either headache or nausea.

